# Linkage Disequilibrium and Evaluation of Genome-Wide Association Mapping Models in Tetraploid Potato

**DOI:** 10.1534/g3.118.200377

**Published:** 2018-08-14

**Authors:** Sanjeev Kumar Sharma, Katrin MacKenzie, Karen McLean, Finlay Dale, Steve Daniels, Glenn J. Bryan

**Affiliations:** * The James Hutton Institute, Dundee DD2 5DA, UK; † Biomathematics and Statistics Scotland (BioSS), Dundee DD2 5DA, UK; ‡ Cygnet PB Ltd, Cambridge, CB21 6AS, UK

**Keywords:** Linkage disequilibrium, Kinship, Population structure, Genome-wide association studies (GWAS), Mixed models, Tetraploid, Potato, Single-nucleotide polymorphism (SNP)

## Abstract

Genome-wide association studies (GWAS) have become a powerful tool for analyzing complex traits in crop plants. The current study evaluates the efficacy of various GWAS models and methods for elucidating population structure in potato. The presence of significant population structure can lead to detection of spurious marker-trait associations, as well as mask true ones. While appropriate statistical models are needed to detect true marker-trait associations, in most published potato GWAS, a ‘one model fits all traits’ approach has been adopted. We have examined various GWAS models on a large association panel comprising diverse tetraploid potato cultivars and breeding lines, genotyped with single nucleotide polymorphism (SNP) markers. Phenotypic data were generated for 20 quantitative traits assessed in different environments. Best Linear Unbiased Estimates (BLUEs) for these traits were obtained for use in assessing GWAS models. Goodness of fit of GWAS models, derived using different combinations of kinship and population structure for all traits, was evaluated using Quantile-Quantile (Q-Q) plots and genomic control inflation factors (λ_GC_). Kinship was found to play a major role in correcting population confounding effects and results advocate a ‘trait-specific’ fit of different GWAS models. A survey of genome-wide linkage disequilibrium (LD), one of the critical factors affecting GWAS, is also presented and our findings are compared to other recent studies in potato. The genetic material used here, and the outputs of this study represent a novel resource for genetic analysis in potato.

Cultivated potato, *Solanum tuberosum* Group *tuberosum* is a highly heterozygous, autotetraploid crop. Due to the many genetic complexities posed by autopolyploidy, most potato genetic studies have been performed at the diploid level using bi-parental populations. Significant recent progress has been made in the development of improved algorithms and software for linkage and QTL analysis in autopolyploid crop species (Hackett *et al.* 2014; Hackett *et al.* 2013; Rosyara *et al.* 2016). The genes affecting many key agronomic and economically important potato traits remain undiscovered, despite the availability of dense genetic maps as well as the potato genome sequence and associated genomic resources (Sharma *et al.* 2013; Potato Genome Sequencing Consortium 2011). A major contributory factor is that the genetic locations of many trait QTL are imprecisely determined due to the complex genetic architecture of many traits as well as to the use of highly heterozygous parental lines for QTL studies. Moreover, markers found to be tightly linked to useful trait alleles in biparental crosses often have limited transferability to wider germplasm pools. Moreover, biparental populations display recombination limited to a single meiosis, which hampers the resolution of QTL mapping (Stich *et al.* 2013). The accurate molecular dissection of several complex traits at the tetraploid level across a wide range of potato germplasm using high-throughput molecular marker platforms is a highly desirable goal.

Association mapping studies have expanded significantly in number in crop plants and are gaining importance for studying the genetic architecture of traits of agricultural and adaptive significance. Association mapping offers considerable advantages over use of bi-parental populations, such as enhanced mapping resolution, a greater number of traits that can be analyzed in a single study, as well as an increased likelihood that marker-trait associations discovered are likely to be robust across a wider germplasm pool. This latter benefit raises the possibility of using association mapping panels as ‘training sets’ for the establishment of genomic selection models. Early association mapping attempts in potato were largely confined to candidate gene approaches (Achenbach *et al.* 2009; Gebhardt *et al.* 2004; Li *et al.* 2005a; Malosetti *et al.* 2007; Pajerowska-Mukhtar *et al.* 2009; Urbany *et al.* 2011). Though successful in identifying marker-trait associations, the candidate gene approach has some critical issues such as (1) bias caused by the selection criteria used to choose genes on the basis of information available from previous genetic, biochemical and/or physiological studies (Stich *et al.* 2013), (2) limitation to traits for which the biochemical and molecular basis are known (Hall *et al.* 2010), and (3) it is prone to missing important but unknown genes involved in the target traits (Stich *et al.* 2013). Nevertheless, association mapping using candidate genes has been shown to be a powerful approach for complex trait analysis in potato. Potato association mapping studies using genome-wide markers have been performed (D’hoop *et al.* 2014; D’hoop *et al.* 2008; Fischer *et al.* 2013; Lindqvist-Kreuze *et al.* 2014; Rosyara *et al.* 2016; Schönhals *et al.* 2016; Vos *et al.* 2016). These truly genome-wide association studies (GWAS) have led to significant findings of marker alleles associated with important potato traits, such as tuber shape, flesh color, after cooking darkening and enzymatic browning (D’hoop *et al.* 2014), late blight resistance (Lindqvist-Kreuze *et al.* 2014), starch content (Schönhals *et al.* 2016). Perhaps most notable among these was the important role played by GWAS in identifying the *StCDF1* gene, a circadian clock gene underlying a major-effect QTL for plant maturity (Kloosterman *et al.* 2013).

GWAS requires a detailed understanding and exploration of population structure to minimize false-positive and false-negative associations due to population stratification. In early potato association mapping studies, assessment of population structure and GWAS were performed using ‘conventional’ marker types such as amplified-fragment length polymorphisms (AFLPs) and simple sequence repeats (SSRs) (D’hoop *et al.* 2014; D’hoop *et al.* 2008) and a limited set of single-nucleotide polymorphisms (SNPs) (Achenbach *et al.* 2009; Pajerowska-Mukhtar *et al.* 2009). However, for a comprehensive analysis the use of high-throughput and marker-dense genotypic data are preferable. The first such platform in potato containing 8303 SNPs (Felcher *et al.* 2012; Hamilton *et al.* 2011) has been deployed to characterize diploid and tetraploid germplasm (Hardigan *et al.* 2015; Hirsch *et al.* 2013; Kolech *et al.* 2016; Stich *et al.* 2013). A further challenge for performing genetic studies at the tetraploid level has been the lack of suitable automated methods for correctly inferring marker allele dosage. SNPs are co-dominant markers and methods for calling allele dosage in autotetraploids have recently been developed (Hackett *et al.* 2013; Voorrips *et al.* 2011; Schmitz Carley *et al.* 2017). These developments coupled with the potential for performing high-throughput assays have rendered SNPs as the preferred markers for GWAS. Association studies in potato utilizing high-throughput genome-wide markers are increasing (Kloosterman *et al.* 2013; Lindqvist-Kreuze *et al.* 2014; Rosyara *et al.* 2016; van Eck *et al.* 2017; Vos *et al.* 2016; Berdugo-Cely *et al.* 2017; Schmitz Carley *et al.* 2017).

We have developed an association mapping panel of 341 tetraploid potato cultivars and breeding lines, largely comprising European founder and cultivated germplasm but including 29 non-European cultivars. Here, we report the genotyping of this panel using an 8k SNP array and the examination of various GWAS models using a set of 20 quantitative traits. We also present an assessment of genetic diversity, individual relatedness, population structure, and a genome-wide survey of linkage disequilibrium (LD) in potato.

## Materials and Methods

### Field Trials and Phenotyping

Field trials were conducted in 2012 and 2013 at two different sites (Cambridge and York, United Kingdom). At each site replicated trials (two replicates) with two nitrogen levels (100 and 200 kg/ha) were conducted according to an alpha design, thereby leading to a total of eight ‘environments’. Twenty quantitative traits were measured, but not all were phenotyped in every environment (Table S1). The traits analyzed were plant height, yield, days to emergence (assessed as days to reach 50% emergence), stolon attachment (1-9, 1 very strong), tuber uniformity (1-9, 9 very uniform), foliage (height to breadth ratio, assessed at ∼80% ground cover but before canopy closure), total number of tubers, tuber dry matter (percentage dry matter based on air-dried and water-immersed weights), eye depth (1-9; 1 deep, 9 shallow), tuber shape (length to breadth ratio), tuber skin brightness (tuber skin texture; 1-9, 9 very smooth), mean number of stems per plant (assessed after full emergence but before the development of secondary stems; main stems only), tubers per stem, tuber flesh color (1-9; 1 white, 9 deep yellow), after cooking blackening (tubers steamed for 20 min and assessed for darkening after 30 min; 1-9, 1 severe), enzymatic browning (darkening of tuber cut surface assessed after 1 hr; 1-9, 1 severe), tuber fry quality (percentage glucose in tubers stored at 6° with CIPC (Chlorpropham) for ∼3 months after harvest), dormancy break (number of tubers with sprouts > 3 mm, storage at 10° without CIPC), ethylene treated tuber sprouting, and maturity. To generate phenotypic values for each trait, genotype was modeled as a fixed effect while all other effects were treated as random, and the Best Linear Unbiased Estimates (BLUEs) for all traits were calculated using REML implemented in Genstat 15^th^ edition (VSN International Limited, http://www.vsni.co.uk). These trait BLUEs were used in GWAS as well as all other analyses involving phenotypic values.

Field trials were rogued by potato field staff for any discrepancies in the observed phenotypes and misidentification/mislabeling of clones. Genotypes which showed inconsistent phenotypes were sent for DNA fingerprinting at Science and Advice for Scottish Agriculture (SASA, https://www.sasa.gov.uk/) for further validation against the varietal SSR genotypic database held at SASA. Correspondence of phenotypic data between replicates, sites and years was also assessed for any gross inconsistencies. For all traits, residuals plots from REML analysis were examined for presence of any outliers and adherence to a normal distribution.

### Germplasm and Genotyping

The germplasm panel comprised 351 diverse tetraploid genotypes including a set of 57 advanced breeding lines. Seed tuber material was obtained from SASA or from stocks held at The James Hutton Institute (Hutton) and multiplied in 2011, the year preceding the first field trial. In addition, we included seven diverse diploid potato genotypes in the genotyping panel to act as ‘outgroups’ and to aid validation of population structure analysis. The diploid material included the sequenced doubled monoploid *Solanum tuberosum* group Phureja DM1-3 516 R44 clone (Lightbourn and Veilleux 2007; Potato Genome Sequencing Consortium 2011), hereafter referred to as DM. The details of the germplasm included in the study are presented in Table S2.

Genomic DNA was extracted from young leaf tissue from individual field grown plants using the Qiagen DNeasy Plant Maxi Kit (Qiagen), quantified using the Quant-iTTM PicoGreen dsDNA Assay Kit (Invitrogen, San Diego, CA), and normalized to a concentration of 30 ng/μL. The panel was genotyped at GenProbe (Liverpool, UK) for the Infinium 8k Potato SNP Array (Felcher *et al.* 2012; Hamilton *et al.* 2011) according to the manufacturer’s protocols. The cultivar Adirondack was ‘spiked’ as a control in all 16 Infinium array BeadChip genotyping batches of 24 genotypes each. SNP genotypes were called using R package fitTetra (Voorrips *et al.* 2011) which employs a mixture model based on the allele signal ratio and fits distributions to the response signals to classify the individuals into one of the five possible genotypic classes in the tetraploid genotype model, giving dosage information (0, 1, 2, 3 or 4) for each SNP. SNPs with inconsistent genotype calls across control tetraploid samples (cv. Adirondack) and those reported as mapping to more than one location in the potato genome (Hirsch *et al.* 2013) were excluded (Table S3). For performing GWAS, SNPs with 20% (or more) missing data and 5% (or below) minor allele frequency (MAF) were excluded. Physical positions for all SNPs were derived from Sharma *et al.* (2013).

### Kinship and Population Structure Analyses

SNP dosage scores were used as input data in the SAS/STAT Distance Procedure for the estimation of Gower’s Similarity Metric (Gower 1967) among all individuals and the estimated matrix was used as a genomic relationship matrix. Population structure was assessed using three different approaches, namely principal component analysis (PCA), non-metric multidimensional scaling (nMDS) and STRUCTURE (STR) analysis. The genotype membership matrices (Q) obtained from the population structure analyses were used as covariates in the GWAS regression models. PCA was performed on the genomic relationship matrix and the first 20 eigenvectors were calculated. For nMDS, the square root of the symmetric relationship matrix obtained via Singular Value Decomposition was used and the analysis was performed with data fitting up to 20 dimensions. PCA and nMDS were performed using JMP Genomics 7.1 software (SAS Institute, Cary NC). Model-based analysis of population structure was carried out using STRUCTURE software (Pritchard *et al.* 2000) with ‘admixture’ model assuming allele frequencies are correlated between inferred populations. The ‘burn-in’ and simulation stages were both set at 10,000 iterations. The number of subpopulations was evaluated from 1 to 10 and each analysis was replicated three times. The optimal population structure estimates (Q matrices) from nMDS (dimensions) and PCA (eigenvectors) were chosen by evaluating their respective screeplots displaying ‘badness-of-fit’ and eigenvalues, respectively. In STRUCTURE subpopulations were inferred by the *ΔK* method of Evanno *et al.* (2005) and a single replicate from the chosen *K* was used to assign the probability that a genotype belonged to in each subpopulation.

### Genome-wide Association Analysis

GWAS model procedures were as described by Yu *et al.* (2006) implemented in GWASpoly R package (Rosyara *et al.* 2016) using additive model; and follow the linear mixed model equation as expressed below:

y=Xβ+Sα+Qv+Zµ+ε

where **y** is a vector of observed phenotypes; **β** is a vector of fixed effects other than the SNP under testing and/or population structure effects; **α** is a vector of SNP effects; **v** is a vector of population effects where *Q*, modeled as a fixed effect, refers to the incidence matrix for subpopulation covariates relating **y** to **v**; **µ** is a vector of random polygenic background effects with covariance proportional to a kinship matrix *K* with Var[**µ**] = *σ_g_^2^K*; **ε** is a vector of residual effects with Var[**ε**] = *Iσ_e_*^2^; X, S and Z are incidence matrices of 1s and 0s relating y to **β**, **α** and **µ**, respectively; and *σ_g_^2^* and *σ_e_^2^* denote genetic and residual variance, respectively. This mixed model equation was adapted to perform GWAS using four different statistical models, as detailed below:

- Naïve model, without controlling for population structure ‘Q’ or individual relatedness ‘K’
- Kinship model, controlling just for individual relatedness
- Population Structure model, controlling for population structure effects
- Full model, accounting for kinship as well as population structure confounding effects

In addition to the description above, all these four models had SNP fitted as a fixed effect and are hereafter referred to as Naïve, K, Q and QK models, respectively. Q and QK models were successively evaluated using population structure estimates derived from three different methods as described in the preceding section, thereby leading to a total of eight GWAS models being tested. Fitness of different GWAS models for all traits was evaluated using Quantile-Quantile (Q-Q) plots of the observed *vs.* expected –log10(*p*) values which should follow a uniform distribution under the null hypothesis. Models were ranked using genomic control inflation factor (λ_GC_) metric calculated using R package ‘GenABEL’ (GenABEL project developers 2013). Bonferroni correction (with genome-wide α = 0.05) was used for establishing a p-value detection threshold for statistical significance.

### Linkage Disequilibrium

Pearson correlation coefficient (r^2^) was used to calculate correlations between marker-pairs using SNP dosage scores (0 to 4). LD was calculated based on marker pairs located within (a) chromosomal short arm, (b) chromosomal long arm, (c) euchromatin (both chromosomal arms combined), (d) heterochromatin, and (e) whole chromosomal region for all 12 chromosomes. Extent of LD decay was estimated by implementing Quantile regression (R package ’quantreg’; Koenker 2017) on the 90^th^ percentile as recommended by Vos *et al.* (2017). From the fitted regression two LD estimators were obtained, *viz*. LD_1/2max,90_ and LD_1/10,90_, denoting the distances at which LD equals one-half of its maximum fitted r^2^ value (r^2^_max,90_) and where r^2^ equals 0.1 on the 90^th^ percentile, respectively. For whole chromosomal regions, extent of LD decay was also estimated using non-linear regression of r^2^ against the physical map distance according to Marroni *et al.* (2011). Marker positions for the different subsets of the genome were derived from Sharma *et al.* (2013).

### Genetic Diversity

The measure of polymorphic information content (PIC) for each SNP was calculated according to Botstein *et al.* (1980). Pairwise genetic distances were calculated using Nei’s (1972) distance estimate which were further subjected to hierarchical clustering using ward.d2 method (Murtagh and Legendre 2014). Trees were constructed using R package dendextend.

### Data availability

Supplemental Material is available at figshare and brief captions for the uploaded material are provided here, as follows: Table S1 (traits phenotyped); Table S2 (germplasm included in the study); Table S3 (List of SNPs mapping to multiple positions); Table S4 (GenomeStudio and fitTetra genotype call comparisons); Table S5 (Chromosomal region-wise details of SolCAP SNPs); Table S6 (Size of pericentromeric heterochromatin); Table S7 (Pairwise measures of population differentiation); Table S8 (Significant marker-trait associations); Table S9 (Annotation of SolCAP SNPs used in the study); Table S10 (Genotype and phenotype data); Figure S1 (Manual and automated SNP genotype calling example); Figure S2 (PCA performed on the genomic relationship matrix of the association panel clones); Figure S3 (Traits Pairs plot); Figure S4 and S5 (LD in chromosomal short and long arms, respectively); Figure S6 (nMDS Screeplot); Figure S7 (population structure present in the association panel); Figure S8 (clustering of genotypes based on Nei (1972) genetic distance); Figure S9 (PCA Screeplot); Figure S10 (STRUCTURE results); Figures S11 and S12 (nMDS and PCA based population stratification); Figures S13 and 14 (Q-Q plots for different categories of model comparisons). Supplemental material available at Figshare: https://doi.org/10.25387/g3.6716033.

## Results and Discussion

### Germplasm and Genotyping

Germplasm included in the study, including geographic origin, year of introduction, and predominant market class, is listed in Table S2. The tetraploid panel and selected diploid material were genotyped using the 8303 Illumina Infinium SNP array (Felcher *et al.* 2012). fitTetra analysis resolved genotypic classes for 6059 SNPs. Genotyping accuracy was assessed by comparing genotype scores among 16 control (cv. Adirondack) samples. Genotype calls were largely in agreement with a consistency rate of 98.3%. SNPs displaying inconsistent genotyping calls across control samples as well as those with multiple mapping locations in the potato genome were excluded yielding a set of 5,718 robust SNPs for further analyses. Illumina standard SNP calling software GenomeStudio, at the time of analysis, was not suitable for automatic genotype calling in tetraploids. Genotype score comparisons (for tetraploids) from these platforms largely corroborated each other (data not shown) and a representative example is shown for the marker ‘solcap_snp_c1_1000’ (Figure S1). A comparison of genotype scorings from both approaches for all diploid genotypes is presented in Table S4.

Genotypic data were further verified by the correspondence of the clusters obtained using PCA with known information about the germplasm. The very tight clustering of 16 control ‘Adirondack’ samples further demonstrated genotyping data integrity with negligible or very low level of technical variation across the 16 genotyping batches. The remainder of the tetraploid clones clustered into a cohesive group while the diploid clones formed three distinct groups, the sequenced Group Phureja clone DM clustering with the closely related Group Phureja diploid cultivar ‘Mayan Gold’ as expected (Figure S2).

**Figure 1:**
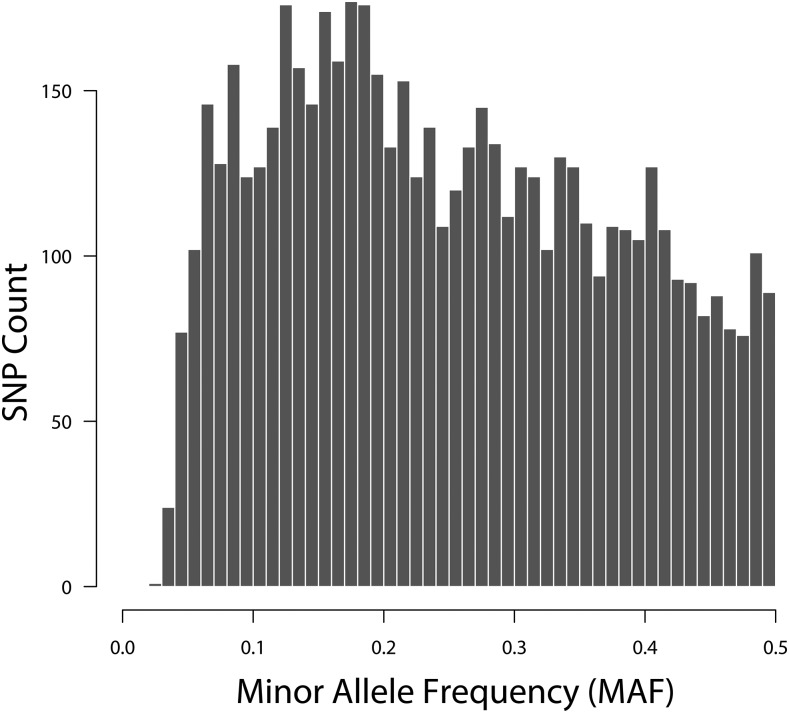
Minor Allele Frequency (MAF) distribution of 5,718 SolCAP SNPs in 341 tetraploid genotypes.

**Figure 2:**
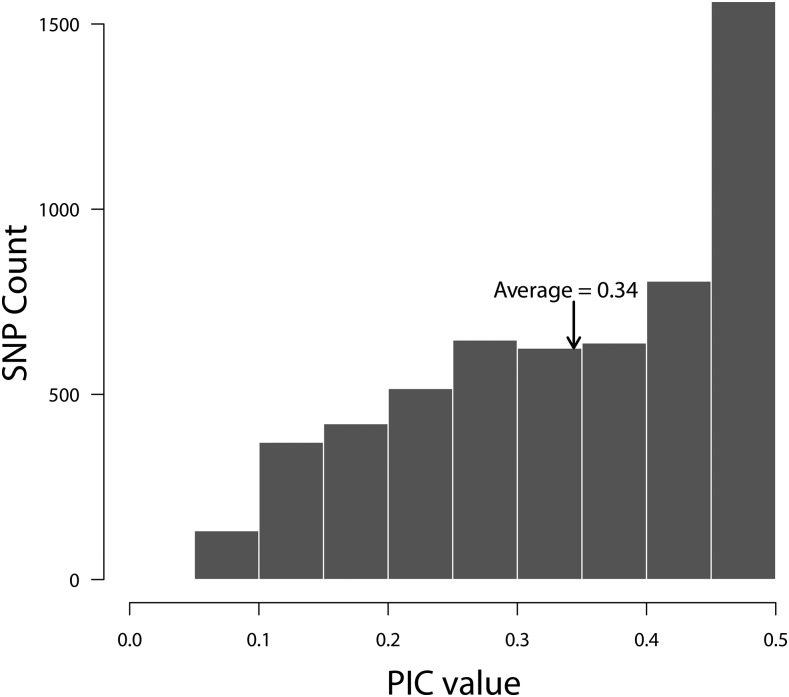
Distribution of polymorphic information content (PIC) values calculated for 341 tetraploid genotypes across 5,718 SolCAP SNPs.

The germplasm panel displays a broad range of genotypic and phenotypic diversity, reflecting the diverse geographic origins, release dates, and market classes of the selected potato varieties and breeding clones. Distribution of minor allele frequencies (MAF) across all tetraploid genotypes is shown in Figure 1. The genotype panel showed enrichment for common SNPs with higher MAF (>0.1) values, desirable for performing GWAS. Genetic diversity in the panel was assessed using estimates of marker PIC values. The PIC values of the SolCAP SNPs for the association panel ranged from 0.06 to 0.50 with a mean value of 0.34 (Figure 2). Stich *et al.* (2013) reported a similar average PIC value of 0.35 in a study involving cultivated European tetraploid material genotyped with the same panel. The SolCAP SNP platform is derived using SNPs from only six potato cultivars (Hamilton *et al.* 2011), which could possibly cause ascertainment bias and limit its applicability in analyzing the germplasm which is not well represented by these six cultivars. However, in the current analysis the majority of SNPs were highly polymorphic with 63.5% showing PIC values above 0.35 and none falling below 0.05 (Figure 2). The observed PIC values indicate high levels of polymorphism in European potato cultivars further validating the suitability of the deployed SNP platform for genetic studies on cultivated potato. Among the six cultivars used in developing the SNP platform, only one (Bintje) is from Europe, the remainder being North American. Potato is believed to have been introduced into North America from Europe (Love 1999), which explains the good representation and sharing of SNP alleles between European and North American cultivars. Moreover, Uitdewilligen *et al.* (2013) reported that more than half of the common SNPs identified in a panel of 83 tetraploid potato cultivars were discovered by sequencing any three random cultivars. Given its history the SNP array used here may well display ascertainment bias for a very diverse set of potato germplasm including non-cultivated material however the inclusion of six diverse cultivars in its design makes it eminently suitable for genotyping a wide cultivated potato population as in this study.

### Phenotyping

**Figure 3:**
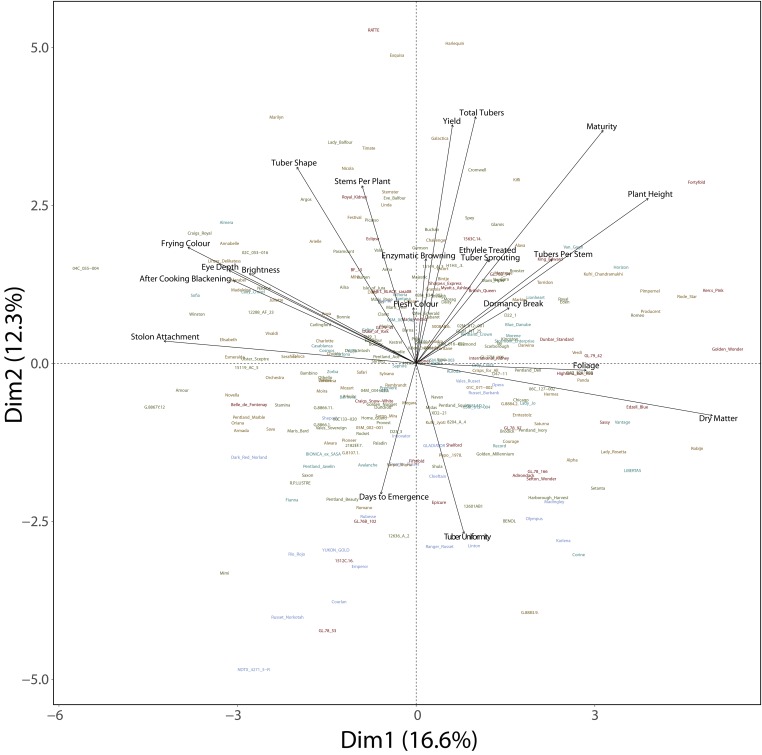
Biplot of the first two significant components obtained by applying PCA to the BLUEs of 20 traits for 290 tetraploid clones. Genotypes are colored according to the nMDS-based subpopulations.

The association panel was phenotyped for 20 quantitative traits in up to eight environments (Cambridge Nitrogen plus-2012, York Nitrogen plus-2012, Cambridge Nitrogen minus-2012, York Nitrogen minus-2012, Cambridge Nitrogen plus-2013, York Nitrogen plus-2013, Cambridge Nitrogen minus-2013, York Nitrogen minus-2013). A ‘pairs’ plot showing a scatterplot of the relationship between traits, the distribution of individual traits and the correlation between traits is presented in Figure S3. The residuals for all traits were normally distributed (data not shown). Figure 3 shows a biplot for the first 2 dimensions for the PCA performed on the full set of 20 traits. The first two axes account for 17.4% and 12.8% of the variation in the data. The colors denote the subpopulations identified by nMDS analysis (described in the subsequent sections). Figure 3 clearly shows the effects of breeding for different traits for varied end-uses over the last more than 100 years, for example, dry matter and yield both have high but quite different correlations with the first two principal components. There is no clear or obvious relationship between any of the traits and the nMDS-derived subpopulations.

### Assessment of Genome-wide Linkage Disequilibrium

Linkage disequilibrium in the association panel was estimated using Pearson’s r^2^ statistic using pairwise combinations of SNPs present across all 12 chromosomes. Previously LD in potato has been assessed using a varying level of structural units ranging from a few fragments per chromosome (Simko *et al.* 2006) to genome-wide superscaffolds (Stich *et al.* 2013). A more recent study (Vos *et al.* 2017) estimated short-range LD separately for pericentromeric heterochromatin and chromosomal arms. Here, we have estimated extent of LD in different regions (short and long arms individually as well as combined, and pericentromeric heterochromatin) of each chromosome as well as the whole chromosome. Marker positions in these subsets of the genome were obtained from Sharma *et al.* (2013) and a list of markers included in these specific regions is provided in Table S5.

**Figure 4:**
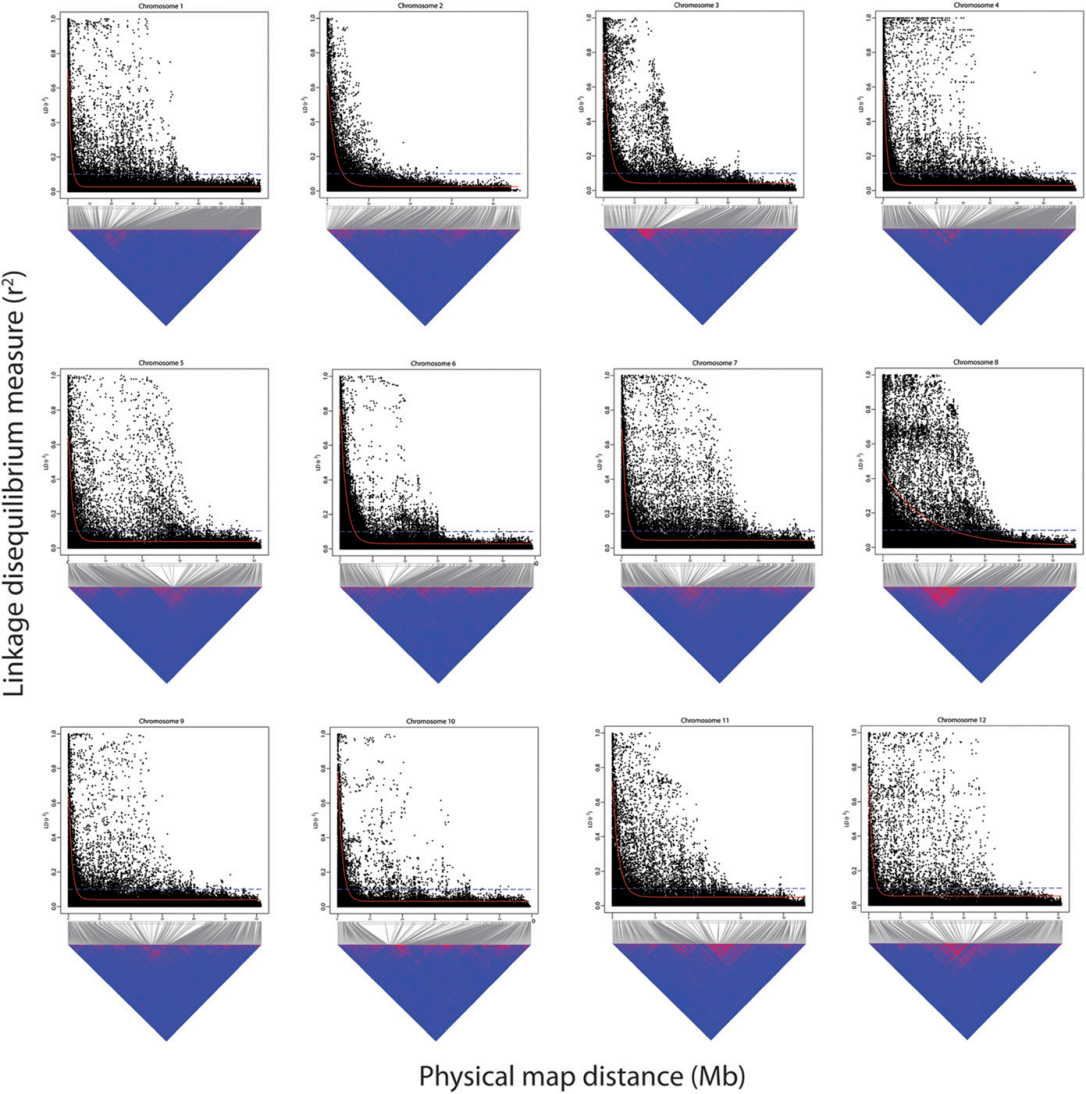
Upper panels: Linkage disequilibrium (LD) measure r^2^ in the association panel plotted *vs.* the physical map distance (Mb) between pairs of SNPs located on the whole chromosomal region for all 12 chromosomes. The trend line of the nonlinear quantile regression of r^2^ (90^th^ percentile) *vs.* the physical map distance between the SNP markers is depicted in red, dashed blue line depicts the standard LD decay threshold (r^2^ = 0.1). Lower panels: Genome-wide LD scans for all 12 chromosomes using 5,718 SNPs. Red areas show regions with higher LD.

**Figure 5:**
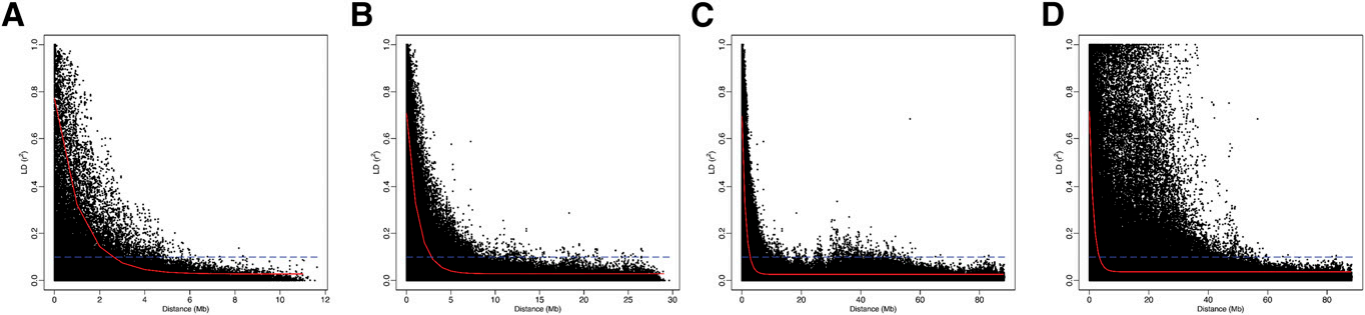
Linkage disequilibrium (LD) measure r^2^ plotted *vs.* the physical map distance (Mb) between pairs of SNPs in the association panel located on (A) chromosomal short arm, (B) chromosomal long arm, (C) both arms combined (euchromatin), and (D) whole chromosome region. The illustrations include combined analyses for all 12 chromosomes except chromosomal short arm for which chromosome 2 is excluded as it is pericentric. The trend line of the nonlinear quantile regression of r^2^ (90^th^ percentile) *vs.* the physical map distance between the SNP markers is depicted in red, dashed blue line depicts the standard LD decay threshold (r^2^ = 0.1).

The r^2^_max,90_, LD_1/2max,90_ and LD_1/10,90_ estimates for the different subsets of the genome are provided in Table 1. The trends observed among 12 chromosomes displayed a moderate LD decay in potato. Inter-chromosomal differences in LD decay patterns were also visible with each chromosome showing a different physical distance range exceeding which the LD decayed below the most commonly used significant threshold value of 0.1 (D’hoop *et al.* 2010; Stich *et al.* 2013). The minimum and maximum LD_1/10,90_ limits for short and long chromosomal arms were different and also differed by chromosome. However, the overall LD_1/10,90_ for short and long arms and the combined euchromatin were comparable *viz*. 2.52 Mb, 2.76 Mb and 2.73 Mb, respectively. LD decay plots for short and long chromosomal arms for all 12 chromosomes are shown in Figure S4 and Figure S5, respectively. LD decay in the pericentromeric heterochromatin for all chromosomes was negligible due to the suppression of recombination in this fraction of the genome, therefore, it was not possible to model LD decay for pericentromeric heterochromatin at the set thresholds as also reported by Vos *et al.* (2017). Despite this insignificant LD decay in heterochromatin, the overall LD_1/10,90_ calculated at whole chromosome level (3.27 Mb) was within twofold limits of that observed for the euchromatin (2.73 Mb). Chromosome-scale LD blocks and decay patterns for all 12 chromosomes are shown in Figure 4; and combined LD decay plots over all chromosomes for the analyzed subsets of the genome are presented in Figure 5. The extended spread of the points, as compared to the typical L-shaped LD decay patterns, in the higher LD range (Figure 4) closely approximates the magnitude of the pericentromeric heterochromatin boundaries reported for all chromosomes (Sharma *et al.* 2013; Table S6). All chromosomes exhibited modest LD_1/10,90_ at the whole chromosomal-region level, ranging from 2.54 Mb (Chromosome 9) to 4.68 Mb (Chromosome 3), whereas chromosome 8 displayed extremely conserved LD_1/10,90_ of 20.04 Mb. Berdugo-Cely *et al.* (2017) also report slowest LD decay for chromosome 8 (LD_1/10,90_ = 8 Mb) in their autotetraploid Andigena population (N = 652). Figure 4 also shows that large LD blocks on all chromosomes are visible but the one present on chromosome 8 is the most prominent. Inheritance of such large ancestral LD block suggests chromosome 8 has undergone selection pressures which differ from other chromosomes in some as yet unknown way during the last 150 years of breeding. Vos *et al.* (2015) have reported chromosomal positions of new variants introduced since 1945 by introgression breeding. Although the numerical breakdown per chromosome is not provided, from the illustration provided (Figure 5 in Vos *et al.* 2015) it is evident that chromosome 8 has received the least (almost negligible) number of introgressed segments/variants in the last six decades of potato breeding.

**Table 1:** Extent of LD decay in different regions of the 12 chromosomes

Chromosome	Short arm			Long arm			Euchromatin*^a^*			Whole chromosome		
	r^2^_max,90_	LD_1/2max,90_	LD_1/10,90_	r^2^_max,90_	LD_1/2max,90_	LD_1/10,90_	r^2^_max,90_	LD_1/2max,90_	LD_1/10,90_	r^2^_max,90_	LD_1/2max,90_	LD_1/10,90_
1	0.79	0.71	2.56	0.70	0.89	2.66	0.71	0.89	2.66	0.73	0.90	2.78
2	NA	NA	NA	0.65	1.05	3.03	0.65	1.05	3.03	0.72	1.35	4.09
3	0.77	0.82	2.82	0.67	1.19	3.57	0.68	1.14	3.46	0.80	1.38	4.68
4	0.60	1.04	2.82	0.66	0.79	2.32	0.64	0.86	2.46	0.64	0.91	2.64
5	0.65	0.78	2.24	0.72	0.59	1.88	0.67	0.70	2.08	0.65	0.85	2.60
6	0.87	1.24	4.62	0.81	1.02	3.37	0.80	1.11	3.66	0.77	1.44	4.59
7	0.72	0.51	1.64	0.71	0.89	2.73	0.69	0.82	2.52	0.69	0.89	2.88
8	0.84	0.86	2.76	0.70	0.97	2.91	0.74	0.93	2.88	0.43	9.29	20.04
9	0.59	0.68	1.93	0.67	0.86	2.60	0.79	0.67	2.22	0.65	0.83	2.54
10	0.56	1.03	2.92	0.82	0.93	3.06	0.75	0.96	3.03	0.78	0.95	3.10
11	0.81	0.76	2.54	0.53	0.81	2.08	0.77	0.71	2.30	0.72	1.09	3.67
12	0.53	0.72	1.86	0.72	0.71	2.14	0.72	0.66	2.01	0.71	0.86	2.91
All Combined	0.77	0.79	2.52	0.71	0.90	2.76	0.70	0.91	2.73	0.71	1.04	3.27

a Short and long arms combined.

r^2^_max,90_: Maximun Pearson correlation coefficient (r^2^) achieved in the 90^th^ percentile.

LD_1/2max,90_: Physical distance (Mb) at which LD has decayed to half its maximum r^2^ value in the 90^th^ percentile.

LD_1/10,90_: Physical distance (Mb) at which LD has decayed to r^2^ = 1/10 in the 90^th^ percentile.

NA, not applicable.

Discrepancies in LD estimates could arise from differences in the type of populations under examination, spacing and type of markers used, genotyping methods, physical/genetic distance units covered and the significance thresholds used for defining the LD decay. For these reasons a direct comparison of LD patterns and estimates observed in previous potato studies is not feasible. However, overall LD trends observed in different studies can still be compared for drawing broad inferences. D’hoop *et al.* (2010) and Simko *et al.* (2006) reported decay of LD to r^2^ equalling 0.1 within a distance of 5 cM and 10 cM, respectively. In our study, overall LD_1/10,90_ for euchromatin and whole chromosomal regions were 2.73 Mb and 3.27 Mb, respectively. Relating physical size of the assembled potato genome (727 Mb; Potato Genome Sequencing Consortium 2011) and only the euchromatin (∼398 Mb, Table S6; Sharma *et al.* 2013) to a representative genetic map in the order of 754 cM (Prashar *et al.* 2014), 1 Mb corresponds to about 1 cM and 2 cM using the respective scales of the genome. Considering these estimates, the extent of LD decay observed in our study is comparable to those reported by D’hoop *et al.* (2010) and Simko *et al.* (2006). Recently Vos *et al.* (2017) suggested LD_1/2max,90_ as an unbiased estimator for comparing extent of LD decay across different studies. The implementation of the same estimator in the current study facilitates direct comparison of LD decay estimates with those obtained by Vos *et al.* (2017). These authors studied LD decay rates for different age (year of release) potato groups and reported a decline in LD over the last century from an LD_1/2max,90_ of 1.5 Mb in cultivars released before 1945 to 0.6 Mb in the recent cultivars released after 2005. The share of cultivars for these two age categories in their panel was 8.4% and 47.5% respectively as compared to 10% and 12% for the same categories in our association panel. Considering the minority of the cultivars from the latter category, the extent of LD decay (overall euchromatin LD_1/2max,90_ = 0.91 Mb; Table 1) observed in our collection of genotypes corroborates well and lies within the range of LD for different age groups as reported by Vos *et al.* (2017). These authors also conclude that in general background levels of LD are reached at a physical distance ranging from 2 to 4 Mb which are also comparable to those obtained here.

Stich *et al.* (2013) reported a sharp LD decay to an r^2^ value of 0.1 within a distance of 275 bp. This is in steep contrast to the findings observed in the current study as well as to all other previous reports in potato. Potato is propagated vegetatively and cultivars are not separated from their progenitors by many meiotic generations, for example, the maximum number reported by Gebhardt *et al.* (2004) and D’hoop *et al.* (2008) was 5 and 10 respectively. This peculiar feature present in potato also contradicts the very rapid decay in LD observed by Stich *et al.* (2013). Vos *et al.* (2017) attributed contrasting LD estimates reported by Stich *et al.* (2013) to their use of average r^2^ as an LD estimator combined with non-linear regression. In addition to the LD estimators described above, we also estimated LD decay using average r^2^ and non-linear regression (data not shown) as implemented by Stich *et al.* (2013). Since LD estimates by these authors were derived from all genomic superscaffolds (euchromatin as well as heterochromatin) using the same SNP array, the chromosome-scale LD estimates obtained here using the alternative method as described above can be compared to the results reported by Stich *et al.* (2013). For our panel overall chromosome-scale LD_1/10_ was 1.09 Mb which despite being derived from average r^2^ fitted using non-linear regression is still within reasonable LD decay limits expected for potato but much slower than that reported by Stich *et al.* (2013). These observations in combination with Vos *et al.* (2017), derived from two different measures of LD estimation, contradict the claims made by Stich *et al.* (2013) and further suggest the discrepancy in the latter study could be due to the use of a highly diverse and much smaller (N = 36) set of tetraploid genotypes as compared to a larger collection of genotypes deployed in the other studies.

### Analysis of Kinship and Population Structure

Genotyping was carried out for 351 tetraploid and 7 diploid potato genotypes. Initial data quality checks revealed ten pairs of tetraploid genotypes showing more than 99% similarity. These genotypes also contained those clones which during the roguing of field trials had shown morphological features incompatible with those expected from previous knowledge. The identical pairs were DNA fingerprinted with SSRs and only the individuals, one per pair, for which phenotypic assessments and fingerprinting patterns matched with the potato database were retained, thereby, reducing the net number of genotypes in the tetraploid panel from 351 to 341.

**Figure 6:**
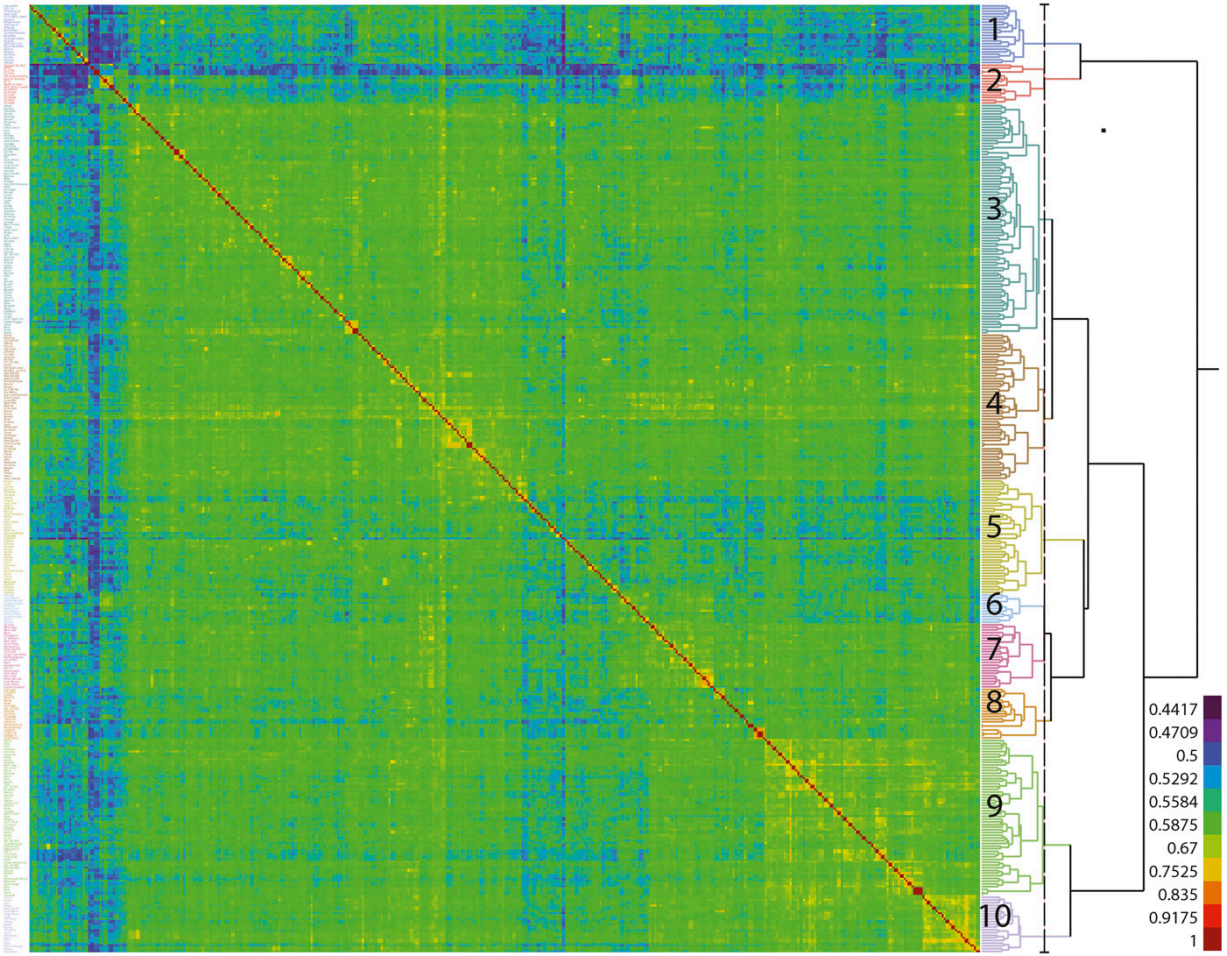
Heatmap displaying relationships among the 341 lines present in the association panel. The red diagonal represents perfect relationship of each line with itself; the symmetric off-diagonal elements represent relationship for pairs of lines. The blocks of warmer colors on the diagonal show clusters of closely related lines. The adjoining dendrogram illustrates Kinship groups identified in the panel. The details of members of these Kinship groups are present in Table S2.

The individual relatedness between genotypes in the revised panel was estimated using a genomic relationship matrix. Kinship analysis assigned more closely related lines such as those belonging to individual breeding programs and/or possessing resistance to specific pest or disease (potato cyst nematode, virus etc.) into ten smaller clusters (hereafter referred to as ‘Kinship groups’). Although the categorization of clones into distinct groups is subjective, the clustering of the panel into ten kinship groups is supported by the hierarchical clustering derived dendrogram (Figure 6). Kinship groups 1 and 2 together are most divergent from the rest of the tetraploid germplasm and quite different from each other. Kinship group 1 largely comprises cultivars from the USA and Canada, whereas group 2 is mainly a collection of ‘salad’ and early potato varieties from various European countries. Regarding the other groups which show quite weak differentiation overall, Kinship groups 3-5 are quite similar comprising mainly Dutch, UK and German cultivars. Kinship group 6 is a small collection of mainly very old UK ‘heritage’ cultivars, as well as two ‘russetted’ US cultivars. Kinship groups 7 and 8 predominantly comprise older UK potato varieties, with groups 9 and 10 being mainly more modern UK and Irish cultivars and breeding lines carrying potato cyst nematode and late blight resistance.

**Figure 7:**
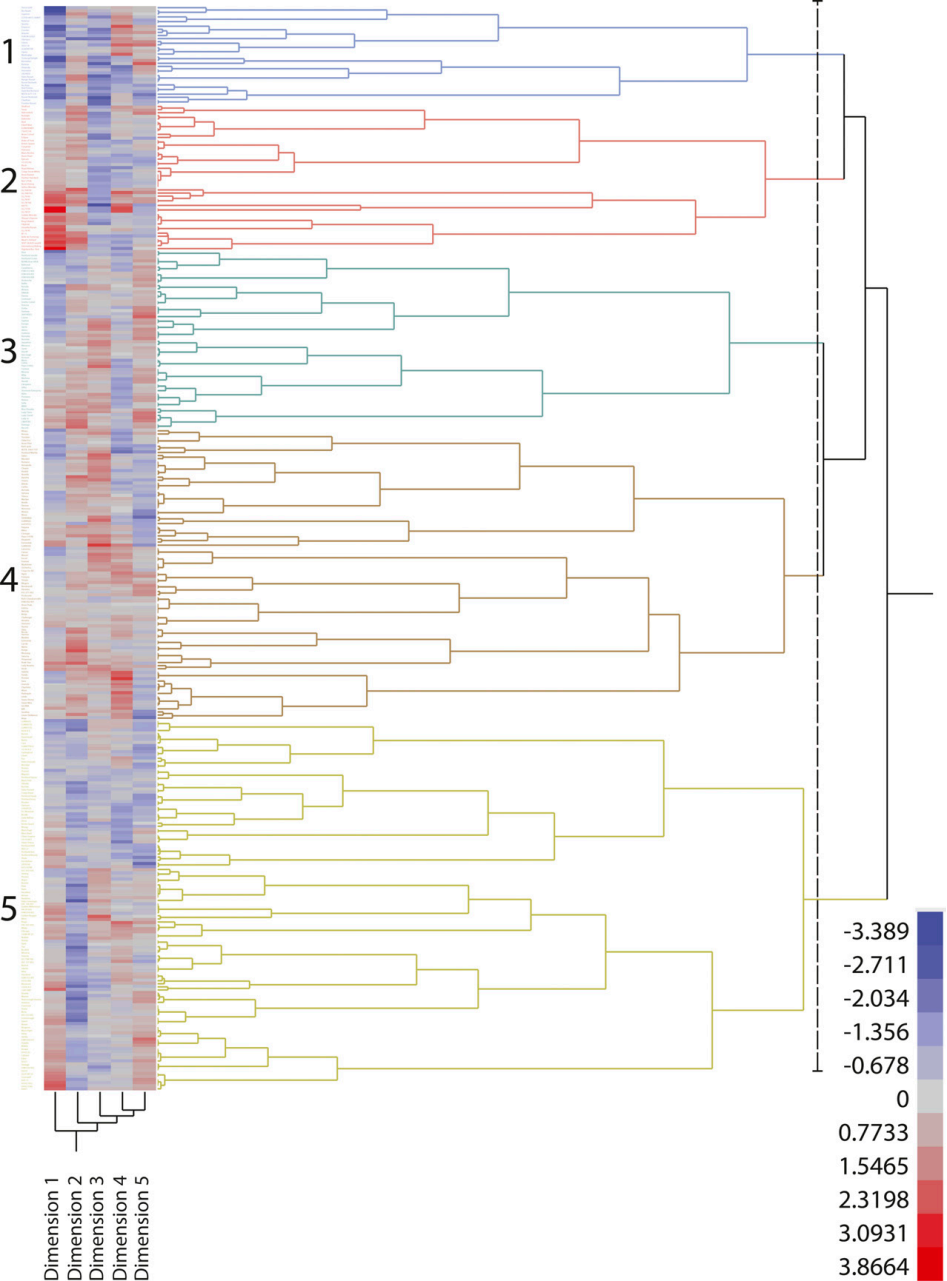
Heatmap and hierarchical clustering of the association panel using a 5-dimension solution from the nMDS (non-metric multidimensional scaling) analysis for adequately representing the panel members (341 clones) in the optimum number of dimensions (subpopulations). The details of members of the identified five subpopulations are present in Table S2.

The level of population stratification was examined using a number of nMDS solutions ranging from 1 to 20 dimensions. The Screeplot in Figure S6 illustrates the badness-of-fit measures for fitting different nMDS solutions. A 5-dimension solution was deemed closest to the point of inflection (elbow junction) with a criterion of representing the full data set in the smallest possible number of dimensions. At the chosen nMDS solution, there was no clear visual separation of clones into distinct subpopulations indicating a lack of strong population structure within the panel (Figure S7). However, nMDS analysis was still able to detect subtle relationships among the clones, possibly resulting from the weak underlying stratification among genotypes, as depicted in the nMDS-based dendrogram (Figure 7) showing clustering of clones into five subpopulations (Q). The nMDS-based dendrogram revealed two large clusters where one cluster comprised four subpopulations while the other formed a single subpopulation. These five putative subpopulations also showed low levels of population differentiation (*F*st, Table S7) which further supports the absence of any strong population structure in the panel used here. These findings are in accordance with the observations made in previous studies (D’hoop *et al.* 2008; Gebhardt *et al.* 2004; Malosetti *et al.* 2007; Simko *et al.* 2004a; Simko *et al.* 2004b; Stich *et al.* 2013) and further validate the notion that cultivated potato lacks a distinct population structure.

The correspondence of kinship groups to the nMDS-derived subpopulations was very high with six kinship groups nesting into their respective subpopulations as single large blocks. Members of the remaining kinship groups did show their assignment to separate subpopulations but still the allocation was in cohesive blocks of at least three genotypes. This allocation of members of individual kinship groups to more than one subpopulation could be attributed to their shared lineage. The details of individual members belonging to these kinship groups and subpopulations are present in Table S2a. The germplasm was also analyzed using phylogenetic analysis (Nei 1972) by including all tetraploid and diploid genotypes. The clustering of tetraploid clones corroborated well with nMDS-derived subpopulations as illustrated in Figure S8. Some advanced breeding lines (*e.g.*, ‘GL’ series) formed distinct clusters and others were interspersed among the cultivar groups. The four diploid wild genotypes clustered into a separate group, which surprisingly included one tetraploid clone, 3053-18, a ‘late blight R-gene differential’ for the resistance gene R5. This result is likely due to close genetic relationship of 3053-18 to the late blight resistant wild species in this cluster, close relatives of which were used to introgress late blight resistance (Trognitz and Trognitz 2007). The remaining three diploid genotypes (DM, *S. tuberosum* group *Phureja* cv. MayanGold and *S. stenotomum*) were clustered with one of the tetraploid clusters predominantly containing older (mainly salad) cultivars and advanced breeding lines.

Population structure was also assessed using PCA (Figure S9) and STRUCTURE (Figure S10). Population structure obtained using PCA and nMDS was compared to visualize patterns of variation in terms of ‘country of origin’ and ‘year of release’. The assessment using ‘year of release’ failed to reveal any specific patterns for both PCA as well as nMDS (data not shown). This observation contrasts with that of D’hoop *et al.* (2010) who reported subpopulations differing in year of market release in a set of 430 tetraploid cultivars. Stich *et al.* (2013) also did not detect any correlation between population structure and the cultivar release date. They attributed this difference to the lack of enough statistical power in their analysis as their panel comprised only 36 tetraploid cultivars, a weakness that does not apply to the current study.

Visualization of nMDS-based population structure in terms of ‘country of origin’ of each genotype yielded a more harmonious representation of the germplasm as compared to the PCA. The subpopulations were not clearly defined but a country-specific pattern was apparent (Figure S11). One subpopulation (olive green, bottom-most) comprised modern UK cultivars while another subpopulation (red, right-most) contained older UK varieties. Two interspersed subpopulations at the center (cyan and brown) primarily contained ‘mainland’ European germplasm; and the remaining one (blue, upper-left) included mainly international non-European material. The analysis suggests the majority of the UK potato cultivars represent more diverse genetic pools than rest of the European and non-European cultivated potato germplasm with limited admixture. Similarly in other studies, population stratification within a region has been reported. For example, two subpopulations of European winter wheat from multiple national programs (Tommasini *et al.* 2007) and four subpopulations from a collection of US soft winter wheat cultivars with release dates spanning approximately 30 years (Breseghello and Sorrells 2006) were previously reported. In contrast to nMDS, no region-based grouping patterns were visible in clusters generated using first two PCA eigenvectors (Figure S12). Previous studies have also shown nMDS as a more appropriate method for visualizing population structure as it can capture the underlying population stratification with the first few dimensions that may not be inferred with the same number of eigenvectors using PCA or principal co-ordinates analysis (PCO) (Miclaus *et al.* 2009).

### Evaluation of Population Structure Estimation Methods for GWAS

All GWAS analyses described here as well as in the subsequent sections were performed on 290 tetraploid clones. This reduction in the association panel was due to clone identity issues, as described in the preceding section, as well as loss of some genotypes due to lack of enough seed tubers for successive field trials. Seed tubers for these genotypes were not supplemented from other sources to avoid inducing any bias arising due to variation in seed tuber generation as the seed material for the reported study was generated from a designated seed production site in the preceding year. Regarding markers, a total of 5,157 SNPs were retained for GWAS after filtering for missing data (>= 20%) and minor allele frequency (MAF=<0.05) thresholds. Performance of GWAS models was evaluated using Q-Q plots of the expected *vs.* observed –log_10_(*p*) values and genomic control inflation factors (λ_GC_) achieved for each ‘trait x model’ combination. The success (ranking) of GWAS models in controlling the overall genome-wide type I error rate (*i.e.*, keeping the genomic control inflation factor to the desired level of λ_GC_∼1), summarized over 20 traits, is shown in Table 2.

**Table 2:** Ranking of GWAS models derived using Genomic Control Inflation Factor (λ_GC_) metric summarized over 20 traits

GWAS Models*^a^*	Ranking*^b^*			
	First	Second	Third	Fourth
**(A) Q Models**				
_nMDS_Q	6	11	3	—
_PCA_Q	6	2	12	—
_STR_Q	8	7	5	—
**(B) Principal Models**				
Naïve	0	0	1	19
K	7	13	0	0
Q	0	0	19	1
QK	13	7	0	0

a nMDS, non-metric multidimensional scaling; PCA, principal component analysis; STR, STRUCTURE.

b Rows and columns under each GWAS model category add up to the total number (20) of traits analyzed.

Various methods have been used for controlling the confounding effects of population structure for GWAS. Here we examined GWAS models with population structure estimates (Q matrices) from three different methods *viz*. STRUCTURE, principal component analysis and non-metric multidimensional scaling. Table 3 displays trait-wise λ_GC_ estimates for Q and QK GWAS models involving Q matrices from above three methods and their overall rankings are provided in Table 2. In Q models, STRUCTURE was marginally more effective (8 traits) in controlling *p*-value inflation than nMDS and PCA while the latter two performed better for 6 traits each. Evaluation of these models as QK models (*i.e.*, with correction for population structure as well as kinship) controlled the *p*-value inflation observed in Q models with different methods for population structure estimation and brought λ_GC_ close to 1. This further implies that with the inclusion of correction for kinship effects, the method used for population structure estimation is not very critical for performing GWAS in potato. Figure S13 and Figure S14 illustrate Q-Q plots obtained from Q and QK models tested using three different Q-matrices, respectively. Overall the results indicate that no single method was able to capture population confounding effects better than the others for all 20 traits. Nevertheless, when overall model performance was taken into consideration, nMDS seemed to provide slight advantage over PCA and STRUCTURE (Table 2A) and was selected for all subsequent assessment of GWAS models.

**Table 3:** Genomic Control Inflation Factor (λ_GC_) analyses of GWAS models as a function of three different methods*^a^* for adjusting population structure

Q only models	λ_GC_			Model Ranking*^b^*		
Trait	_nMDS_Q	_PCA_Q	_STR_Q	_nMDS_Q	_PCA_Q	_STR_Q
After Cooking Blackening	1.569	1.586	1.571	1	3	2
Brightness	1.823	2.105	1.785	2	3	1
Days to Emergence	1.286	1.378	1.244	2	3	1
Dormancy Break	1.086	1.25	1.186	1	3	2
Dry Matter	2.453	2.45	2.494	2	1	3
Enzymatic Browning	1.388	1.613	1.457	1	3	2
Ethylene treated tubers sprouting	1.59	1.562	1.519	3	2	1
Eye Depth	1.666	1.481	1.578	3	1	2
Flesh Color	1.922	3.062	1.966	1	3	2
Foliage	1.168	1.302	1.132	2	3	1
Frying Color	2.254	2.4	2.244	2	3	1
Maturity	1.449	1.428	1.622	2	1	3
Plant Height	1.554	1.692	1.426	2	3	1
stems per Plant	1.444	1.474	1.297	2	3	1
Stolon Attachment	1.678	1.289	1.601	3	1	2
Total Tubers	1.404	1.991	1.319	2	3	1
Tuber Shape	1.888	2.035	2.075	1	2	3
Tuber Uniformity	1.593	1.548	1.621	2	1	3
Tubers per Stem	1.629	1.572	1.653	2	1	3
Yield	1.693	1.86	1.723	1	3	2

QK models*^c^*	λ_GC_					
Trait	_nMDS_QK	_PCA_QK	_STR_QK			
After Cooking Blackening	0.944	0.977	0.956
Brightness	0.906	0.91	0.92
Days to Emergence	0.991	0.975	0.976
Dormancy Break	0.97	0.974	1.003
Dry Matter	0.925	0.953	0.931
Enzymatic Browning	0.945	0.964	0.947
Ethylene treated tubers sprouting	0.971	0.991	0.955
Eye Depth	0.977	0.908	0.93
Flesh Color	0.878	0.861	0.9
Foliage	0.964	0.991	0.927
Frying Color	0.957	0.993	0.969
Maturity	0.928	0.943	1.001
Plant Height	1.026	1.086	1.037
stems per Plant	0.97	0.988	0.965
Stolon Attachment	0.968	0.954	0.951
Total Tubers	0.972	0.949	0.914
Tuber Shape	0.975	0.968	0.981
Tuber Uniformity	1.007	1.025	1.021
Tubers per Stem	0.956	0.969	0.952
Yield	0.978	1.011	0.982

a nMDS, non-metric multidimensional scaling; PCA, principal component analysis; STR, STRUCTURE.

b 1, first; 2, second; 3, third.

c QK models not ranked as inclusion of K in GWAS models brought λGC close to 1 for all three Q methods tested.

### Evaluation of Different GWAS Models

**Figure 8:**
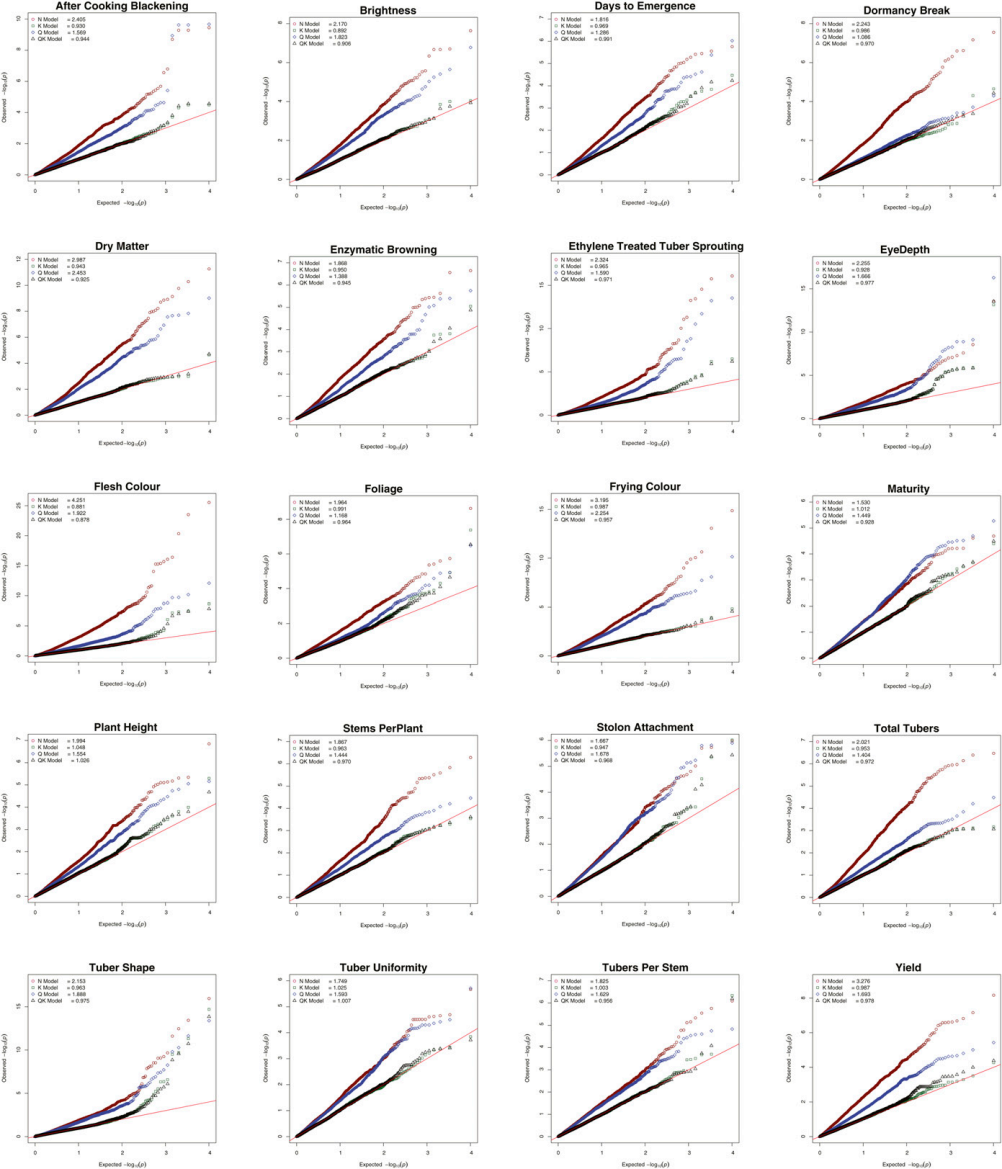
Q-Q plots comparing the inflation of *p*-values for the four principal GWAS models for all 20 traits using the additive marker model. Red circles: Naïve model; Green squares: K model; Blue diamonds: Q model; and Black triangles: QK model. Red line indicates *p*-values under the expected normal distribution.

Four principal GWAS models (Naïve, K, Q and QK) were investigated for all 20 traits. The λ_GC_ values and rankings for these four models achieved for all 20 traits are presented in Table 4. Combined ranking summary for main GWAS models is provided in Table 2B. The efficiency of K and QK models, in keeping *p*-values close to the expected (λ_GC_∼1), were comparable where K model was most effective for 7 traits and QK for 13 traits. Furthermore, comparison of the individual components of the QK model revealed that correction for Kinship was more efficient in overcoming the germplasm confounding effects than adjusting for population structure. As compared to K Model, Q Model never ranked first or second for any of the 20 traits and performed close to K and QK models for only one trait (Dormancy break, Table 4). Q model performed better (ranked third) than Naïve model for all but one trait (Stolon attachment) and λ_GC_ for both models were always inferior (larger) to the K model. Figure 8 displays Q-Q plots obtained for four principal GWAS models for all 20 traits. These plots further reveal that Q-Q plots for K and QK models were largely indistinguishable for all traits and wherever one model performed better than the other, it only improved the analysis marginally. This further implies that kinship alone is sufficient to control type I error (false positives) for conducting GWAS in cultivated potato. Also, the ineffectiveness of Q model in controlling *p*-value inflation further confirms lack of significant population structure in potato.

**Table 4:** Genomic Control Inflation Factor (λ_GC_) analyses of four principal GWAS models

Trait	λ_GC_				Model Ranking*^a^*			
	Naïve	K	Q	QK	Naïve	K	Q	QK
After Cooking Blackening	2.405	0.93	1.569	0.944	4	2	3	1
Brightness	2.17	0.892	1.823	0.906	4	2	3	1
Days to Emergence	1.816	0.969	1.286	0.991	4	2	3	1
Dormancy Break	2.243	0.986	1.086	0.97	4	1	3	2
Dry Matter	2.987	0.943	2.453	0.925	4	1	3	2
Enzymatic Browning	1.868	0.95	1.388	0.945	4	1	3	2
Ethylene treated tubers sprouting	2.324	0.965	1.59	0.971	4	2	3	1
Eye Depth	2.255	0.928	1.666	0.977	4	2	3	1
Flesh Color	4.251	0.881	1.922	0.878	4	1	3	2
Foliage	1.964	0.991	1.168	0.964	4	1	3	2
Frying Color	3.195	0.987	2.254	0.957	4	1	3	2
Maturity	1.53	1.012	1.449	0.928	4	2	3	1
Plant Height	1.994	1.048	1.554	1.026	4	2	3	1
stems per Plant	1.867	0.963	1.444	0.97	4	2	3	1
Stolon Attachment	1.667	0.947	1.678	0.968	3	2	4	1
Total Tubers	2.021	0.953	1.404	0.972	4	2	3	1
Tuber Shape	2.153	0.963	1.888	0.975	4	2	3	1
Tuber Uniformity	1.749	1.025	1.593	1.007	4	2	3	1
Tubers per Stem	1.825	1.003	1.629	0.956	4	2	3	1
Yield	3.276	0.987	1.693	0.978	4	1	3	2

a 1, first; 2, second; 3, third; 4, fourth; for cases where λ_GC_ was deflated (below 1), models closest to ‘1’ were ranked highest.

A careful assessment of kinship and population structure in GWAS studies is required to avoid spurious associations arising from systematic differences in allele frequencies due to the difference in sample ancestries. However, the results described above indicate that this may only be critical when the trait in question is also confounded with the kinship and/or population structure present in the germplasm. Overcompensation of mixed models for population structure and relatedness can lead to false negatives (type II error) as also reported by Zhao *et al.* (2011) in rice. In a study involving evaluation of GWAS models for 13 traits, Rosyara *et al.* (2016) report a preference for the QK model, however, trait specific model fitness details are not divulged. Results here instruct that a single model type is not suitable for all traits and emphasize the need to carefully select an optimal combination of covariates for each trait to avoid false associations as well as overfitting of the GWAS models. Overall, the results endorse greater significance for correcting kinship than population structure in GWAS for cultivated potato.

### Model Validation Using GWAS Results

**Figure 9:**
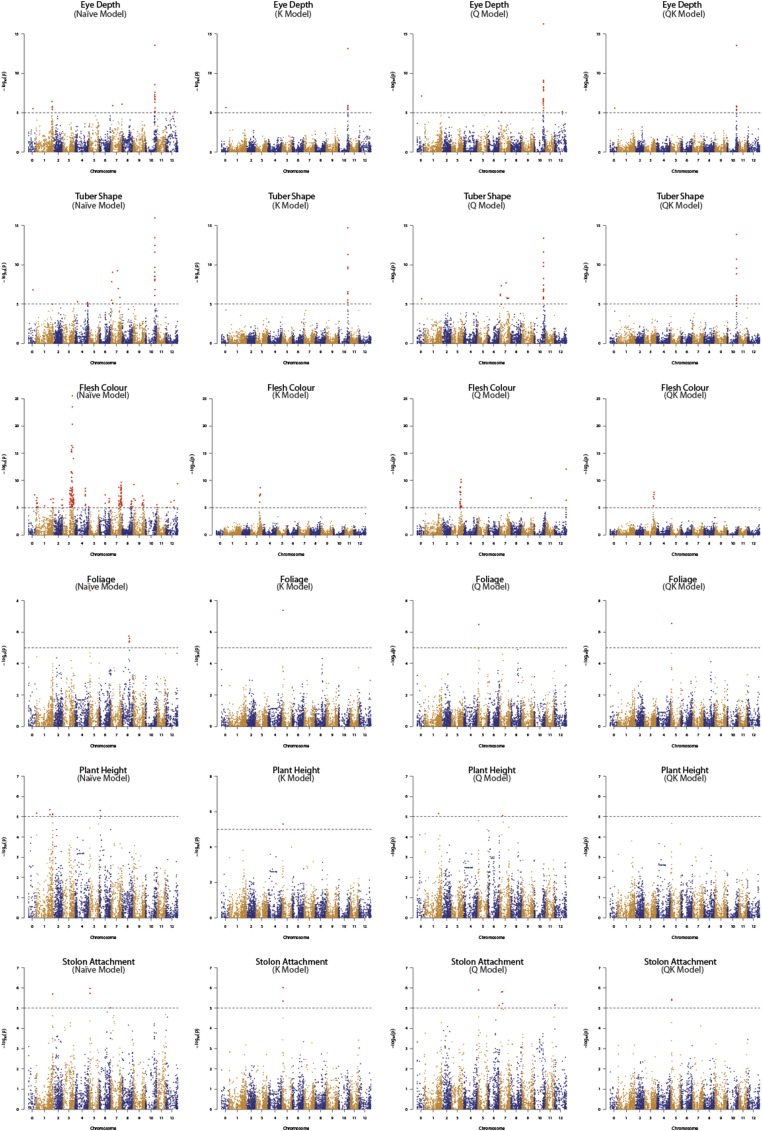
Comparison of Manhattan plots for Naïve, K, Q and QK models. Significance threshold (black dashed line) is based on the genome-wide false positive rate (α = 0.05) for the Bonferroni correction method and the marker-trait associations (MTAs) crossing the set threshold are depicted in large red dots.

We demonstrate here the effectiveness of different models *viz*. Naïve, K, Q and QK for performing GWAS in potato and validate our findings using previously published reports. Figure 9 and Table 5 shows Manhattan plots and significantly associated SNPs identified around well reported QTL regions in potato. Only true marker-trait associations *i.e.*, (a) from models where *p*-value inflation was close to the expected normal value (λ_GC_∼1) and (b) which passed the set Bonferroni correction *p*-value (α = 0.05) threshold are included for reporting GWAS results. Effectiveness of K and QK models over Q and Naïve models in controlling type I error is clearly evident in the Manhattan plots (Figure 9) and the results mainly follow the same pattern as revealed by Q-Q plots (Figure 8) and λ_GC_ values (Table 2). Trait associations obtained from all four main models are listed in Table S8.

**Table 5:** Significant marker-trait associations (MTAs) in the potato GWAS panel

Trait	Marker*^a^*	Chr	Position (bp)	GWAS model	Threshold*^b^*	-log_10_(*p*)
Eye Depth	solcap_snp_c1_8019	10	48863165	K, QK	5	13.15, 13.54
Flesh Color	solcap_snp_c2_20285	3	49393382	K, QK	5	8.67, 7.84
Foliage	solcap_snp_c2_50302	5	5055763	K, QK	5	7.38, 6.54
Plant Height	solcap_snp_c2_50302	5	5055763	K	5	5.3
Stolon Attachment	solcap_snp_c2_50302	5	5055763	K, QK	5	6.02, 5.39
	solcap_snp_c1_14802	5	5051766	K, QK	5	5.35, 5.44
Tuber Shape	solcap_snp_c1_8019	10	48863165	K, QK	5	14.7, 13.85

a Only most significant MTAs are shown; where K and QK did not detect the same SNP as the most significant MTA, the top most MTAs from both models are included.

b *p*-value significance threshold according to Bonferroni correction at 5% genome-wide false positive rate.

GWAS for Tuber Shape and Eye Depth co-identified ‘solcap_snp_c1_8019’ as the most significant association located at 48.9 Mb on chromosome 10. Rosyara *et al.* (2016) also detected the same SNP for these linked traits in their association panel using the same SNP array. Previous biparental mapping studies (Li *et al.* 2005b; Prashar *et al.* 2014; Śliwka *et al.* 2008; Vaneck *et al.* 1994) have also mapped major QTL for Tuber Shape and Eye Depth to the same locus on chromosome 10. Prashar *et al.* (2014) identified ‘solcap_snp_c1_8020’ as the main QTL SNP for this locus which we also report among the most significant associations detected here for Tuber Shape and Eye Depth (Table S8).

Genetic mapping studies have previously reported a major locus affecting plant maturity on chromosome 5 (Bradshaw *et al.* 2008). Analysis of a proxy (for maturity) phenotype ‘Stolon Attachment’ detected a significant association (solcap_snp_c2_50302) on chromosome 5 around 0.5 Mb away from the *StCDF1* gene, identified as the gene underlying the maturity locus by Kloosterman *et al.* (2013). Previous GWAS attempt (Rosyara *et al.* 2016) with the same SNP array did not detect any association for maturity, however a recent study (Schmitz Carley *et al.* 2017) with SolCAP 8K array does identify ‘solcap_snp_c2_50302’ as the most significant association for the maturity locus. Interestingly, analyses of Foliage and Plant Height also revealed the same SNP (solcap_snp_c2_50302) as the most significant association validating the confounded behavior of these traits with maturity.

The *Y*-locus controlling the white-to-yellow flesh color in potato maps to chromosome 3 (Bonierbale *et al.* 1988; Jacobs *et al.* 1995) and is believed to be mainly regulated by the β-carotene hydroxylase (*bch*) gene (Kloosterman *et al.* 2010). Out of the three copies of *bch* present on chromosome 3 (Potato Genome Sequencing Consortium 2011), one isoform (PGSC0003DMG400009501) is located at 44.1 Mb. In this study, a strong association for Flesh Color was observed at 49.4 Mb (solcap_snp_c2_20285) on Chromosome 3 with another significant association at 45.6 Mb (solcap_snp_c2_25653, Table S8), approximately 1.5 Mb away from the causal gene.

Among the GWAS models examined here, Q, K and QK were able to reduce *p*-value inflation relative to the Naïve model, with K and QK further performing significantly better than the Q model. These trends were consistent in λ_GC_ comparison, Q-Q plots and Manhattan plots. K and QK models corroborated well with each other, however, for their co-identified marker-trait associations *p*-values from the K model were generally (4 out of 6) lower and thus more significant than those reported by the QK model (Table 5). Moreover, association for Plant Height was only detected using K model and its absence in the QK model perhaps indicates overfitting of the mixed model. Lack of significant associations as well as appearance of only marginally significant results for some of the traits (Plant Height and Stolon Attachment) could be attributed to low marker density of the SolCAP SNP array as also suggested by Rosyara *et al.* (2016). These authors emphasized the importance of higher marker density and larger population size for future GWAS studies. Here we report that a careful selection of model parameters is equally critical for obtaining true associations and improving success rates of GWAS in potato.

### Implications for GWAS in Potato

The genotypes evaluated here were targeted to diverse end-uses but the population structure analysis did not detect clear separation of cultivars into distinct subpopulations. This is largely expected in potato as breeders do not take population structure into account while choosing parents of a cross. Such practice, however, is reported to generate diverse levels of individual relatedness (Garris *et al.* 2005) as also observed in the examined association panel. The presence of a weak population structure in cultivated potato *per se* could be attributed to admixed populations and also possibly to introgression breeding for developing disease resistance using common resistant parents. Moreover, autotetraploidy and high levels of heterozygosity have a strong tendency to ‘blur’ differences between genotypes and contribute to weak population structuring. Bouaziz *et al.* (2011) compared several methods to counter population stratification for correcting different types of population structure simulated in their study and further opined that addressing populations with a weak population structure requires more careful analysis than with discrete populations. Yu *et al.* (2006) have described five categories to which population members can be assigned. Briefly, these include populations with minimal population structure or familial relatedness (type I), family-based samples (type II), samples with familial relationships within structured population (type III), samples with explicit population structure (type IV) and samples with very high levels of population structure coupled with diverse levels of familial relatedness (type V). It is also implied that while suitable methods for accounting for population structure and individual relatedness need to be implemented to control for false positives (type I error), excessive or unnecessary use of these measures could also lead to loss in statistical power. Considering the description above, the genotypes in our association panel are likely to fall somewhere between type II and III categories as a prominent individual relationship among the panel members was visible accompanied by the presence of weak population stratification. These findings are further supported by the evaluation of different GWAS models where K model performs significantly better than the Q model for all traits and the QK model is only marginally more effective (than K model) for 13 (out of 20) traits. Other studies (Li *et al.* 2011; Rosyara *et al.* 2016; Yu *et al.* 2006) have also reported greater improvement with K model relative to Naïve and Q model implying the efficacy of kinship over population structure in controlling confounding effects due to relatedness.

## Conclusions

The current study analyses LD in various subsets of the genome and reports a moderate level of LD decay in potato. Stich *et al.* (2013), with their estimates of very rapid decay of LD, predict a low success in GWAS using an 8K SNP array on a panel of 300 genotypes and further suggest a requirement of about 3 million SNPs for enhanced success. LD decay estimates provided here suggest the suitability of the 8k array for performing GWAS in potato, although success will also depend on the trait being examined. GWAS with monogenic traits (*e.g.*, tuber shape) with large effect QTL will have greater success with 8k SNP array than with the polygenic traits (*e.g.*, yield) with complex architecture as observed in the current and previous studies (Rosyara *et al.* 2016). Moreover, analyzing pathogen specific resistance traits using 8K array will be challenging due to possible ascertainment bias. Nevertheless, Lindqvist-Kreuze *et al.* (2014) have suitably exploited this array for performing GWAS for late blight resistance in tetraploid potato. Previous GWAS efforts have identified significant associations by targeting candidate loci for the traits of interest (Gebhardt *et al.* 2004; Li *et al.* 2005a; Malosetti *et al.* 2007; Pajerowska-Mukhtar *et al.* 2009; Simko *et al.* 2004a), by using a varying number of random AFLP markers (D’hoop *et al.* 2014; D’hoop *et al.* 2008) and more recently by employing a genome-wide SNP array (Rosyara *et al.* 2016; Schmitz Carley *et al.* 2017; Lindqvist-Kreuze *et al.* 2014). These findings also imply that the number of markers required for performing GWAS in potato may not be as high as predicted by Stich *et al.* (2013), further suggesting that LD decay rates observed in their study are not typical for potato. Moreover, recently genotyping platforms with a larger number of SNPs (Vos *et al.* 2015; Illumina version 3 SNP array) have been developed. These marker-dense platforms would provide for a more accurate correction for different levels of relatedness in GWAS models and will further aid in identifying genomic regions that have been influenced by targeted breeding history for developing cultivars with enhanced traits.

The primary aim of GWAS is to filter real marker-trait associations that arise from physical linkage of loci from spurious effects that result as a consequence of potential confounders such as kinship and population structure. The germplasm examined here shows multiple levels of relatedness with a prominent kinship and weak population structure, despite which the nMDS analysis was able to reveal the underlying patterns of subpopulations in the panel. These subpopulations showed an apparent structuring according to country of origin and further provide novel insights into the breeding strategies adopted in Europe. Evaluation of various GWAS models for 20 traits further demonstrated the importance of applying trait-specific models and exploiting different population structure methods for performing association mapping in potato. The main finding reveals kinship, rather than population structure, as the major factor controlling the level of spurious associations in cultivated potato.
